# Identification of Novel Intronic SNPs in Transporter Genes Associated with Metformin Side Effects

**DOI:** 10.3390/genes14081609

**Published:** 2023-08-11

**Authors:** Natascha Schweighofer, Moritz Strasser, Anna Obermayer, Olivia Trummer, Harald Sourij, Caren Sourij, Barbara Obermayer-Pietsch

**Affiliations:** 1 Division of Endocrinology and Diabetology, Department of Internal Medicine, Medical University of Graz, 8036 Graz, Austria; natascha.schweighofer@yahoo.com (N.S.); moritz.udo.strasser@gmail.com (M.S.); a.obermayer@medunigraz.at (A.O.); ha.sourij@medunigraz.at (H.S.); barbara.obermayer@medunigraz.at (B.O.-P.); 2 Center for Biomarker Research in Medicine, CBmed, 8010 Graz, Austria; 3 Department of Health Studies, Institute of Biomedical, FH Joanneum University of Applied Sciences, 8020 Graz, Austria; 4 Interdisciplinary Metabolic Medicine Trials Unit, Medical University of Graz, 8036 Graz, Austria; 5 Division of Cardiology, Department of Internal Medicine, Medical University of Graz, 8036 Graz, Austria; caren.sourij@medunigraz.at

**Keywords:** metformin, side effects, transporter proteins, single nucleotide polymorphisms, polygenic risk score

## Abstract

Metformin is a widely used and effective medication in type 2 diabetes (T2DM) as well as in polycystic ovary syndrome (PCOS). Single nucleotide polymorphisms (SNPs) contribute to the occurrence of metformin side effects. The aim of the present study was to identify intronic genetic variants modifying the occurrence of metformin side effects and to replicate them in individuals with T2DM and in women with PCOS. We performed Next Generation Sequencing (Illumina Next Seq) of 115 SNPs in a discovery cohort of 120 metformin users and conducted a systematic literature review. Selected SNPs were analysed in two independent cohorts of individuals with either T2DM or PCOS, using 5′-3′exonucleaseassay. A total of 14 SNPs in the organic cation transporters (OCTs) showed associations with side effects in an unadjusted binary logistic regression model, with eight SNPs remaining significantly associated after appropriate adjustment in the discovery cohort. Five SNPs were confirmed in a combined analysis of both replication cohorts but showed different association patterns in subgroup analyses. In an unweighted polygenic risk score (PRS), the risk for metformin side effects increased with the number of risk alleles. Intronic SNPs in the OCT cluster contribute to the development of metformin side effects in individuals with T2DM and in women with PCOS and are therefore of interest for personalized therapy options.

## 1. Introduction

Metformin is the most commonly prescribed drug for the treatment of type 2 diabetes (T2DM) as well as other indications requiring insulin-sensitizing drugs, such as polycystic ovary syndrome (PCOS), presenting with insulin resistance, hyperandrogenemia and female infertility [1]. Recently, metformin has been repurposed and tested for a number of other diseases [2,3,4,5], such as dementia, and also has implications for healthy ageing [6]. Metformin is known to be safe in long term use. Lactic acidosis has been described in people treated with metformin; however, a Cochrane systematic review did not show a significantly increased risk of lactic acidosis with metformin compared to other glucose-lowering drugs [7]. The most commonly reported side effects are gastrointestinal issues such as nausea, vomiting, flatulence, indigestion, abdominal discomfort, heartburn, bloating and diarrhoea. Up to 25% of metformin users suffer from these symptoms, with 5% not able to tolerate metformin at all [8,9]. Furthermore, they strongly decrease persistence of therapy, since up to 48% of metformin users are non-adherent within the first year of metformin intake [2]. Metformin is mainly absorbed by the upper small intestine (20% in the duodenum, about 60% in the jejunum and ileum) and has an absolute bioavailability of 50–60%, with the highest levels in the liver and jejunal sites [3,10]. It is excreted unchanged in urine [3], and about 30% is found in faeces [9]. The main transporters involved in metformin transport are the organic cation transporters (OCTs) and the multidrug and extrusion protein (MATE1). The human OCTs consist of three closely related members: OCT1 (encoded by SLC22A1), OCT2 (encoded by SLC22A2) and OCT3 (encoded by SLC22A3). OCT1 and OCT2 are 70% identical in protein sequence, whereas OCT3 shares 50% sequence homology with OCT1 and OCT2 [11]. In excretory organs, OCTs frequently team up with the MATE1 protein to mediate transepithelial transport of organic cations, encoded by the MATE1 gene also known as SLC47A1. Human MATE1 has only one isoform 570 amino acids in length [12] and is predicted to have 13 transmembrane domains with an extracellular carboxyl terminus and an intracellular amino terminus [13]. GLUT2, encoded by the SLC2A2 gene, is present in the basolateral membrane of enterocytes and of epithelial cells from the kidney where it functions in the second step of transepithelial glucose transport [14]. The intra-individual variability in the efficacy and occurrence of side effects of drugs is highly heritable [15]. Coding SNPs in transport proteins are not only associated with reduced uptake or enhanced elimination, but also with the presence of side effects [8,16,17]. While some non-coding SNPs in the OCT cluster have been described to influence metformin efficacy, data about their involvement in side effect occurrence are scarce [18,19].

The aims of the study were (1) to identify intronic SNPs in genes encoding the OCT cluster that are associated with the occurrence of side effects during metformin use, and to replicate these findings in two cohorts of individuals with disturbances of insulin sensitivity—namely, T2DM and in women with PCOS on metformin medication; and (2) to investigate published metformin-related SNPs in relation to metformin side effects.

## 2. Materials and Methods

### 2.1. Study Populations

#### 2.1.1. Discovery Study

A discovery cohort of 120 metformin users was recruited between September 2016 and 2018 in the Outpatient clinic of the Division of Endocrinology and Diabetology. Participants were included at an age between 18 and 80 years with PCOS or T2DM and metformin therapy for at least 1 month. PCOS was diagnosed according to the Rotterdam criteria [20], and T2DM according to the current guidelines for T2DM [21]. In order to reduce possible confounding factors from critically ill patients, we set the following exclusion criteria. Individuals with GFR < 30, anaemia (haemoglobin < 12 g/dL), more than double the normal value of ASAT (men > 70, women > 60 U/L), ALAT (men > 90, women > 70 U/L), GGT (men > 110, women > 76 U/L); creatinine levels ≥ 1.5 mg/dL (men) or ≥1.4 mg/dL (women) were excluded from the study. Data on medical history, anthropometry and concomitant medications were collected, and metformin intake and side effects were assessed by questionnaires. All participants gave their written informed consent prior to inclusion in the study. All protocol procedures were approved by the local Ethics Committee of the Medical University of Graz (EC-number 26-020 ex 13/14). Anthropometric data: body weight, precise to 0.1 kg, was determined using an electronic scale (model SECA 764, Hamburg, Germany); height, precise to 0.1 cm, was measured using a fixed stadiometer; BMI was calculated as weight in kilograms divided by height in meters, squared (kg/m^2^). Hip and waist were measured according to WHO guidelines (WHO 2012) in centimeters. Definition of metformin side effects: metformin side effects were defined as the report of adverse effects after intake of metformin, regardless of dose, after an initial 1-month run-in period. Concomitant medications: we identified medications reported to inhibit OCTs, proteins that mediate transmembrane trafficking of their target molecules and are required for metformin absorption in the gut as described by Dawed et al. [16]. The use of the following drugs was included as a covariate in binary logistic regression analysis: tricyclic antidepressants (TCAs), proton pump inhibitors (PPIs), citalopram, verapamil, diltiazem, doxazosin spironolactone, clopidogrel, rosiglitazone, quinine, tramadol, codeine, disopyramide, quinidine, repaglinide, propafenone, ketoconazole, morphine, tropisetron, ondansetron, antipsychotic agents and tyrosine kinase inhibitors.

#### 2.1.2. Replication Cohort 1—Individuals with T2DM

Samples from the Graz Diabetes Registry for Biomarker Research (GIRO), a prospective cohort study at the Outpatient Clinics for Diabetes, Lipids and Metabolic Disease at the Division of Endocrinology and Diabetology, including individuals with type 1 diabetes, T2DM, rare types of diabetes like Maturity onset diabetes of the young, type 3 diabetes, obese people undergoing bariatric surgery and patients with lipid metabolism disorder were used for replication. Participants with T2DM on metformin therapy were additionally contacted by phone to collect data about metformin intake and potential side effects.

#### 2.1.3. Replication Cohort 2—Women with PCOS

The second replication cohort consisted of 178 women with PCOS from the local PCOS Cohort Registry, who were treated with metformin for at least 3 months. The women were routinely referred to our Outpatient Endocrinology Clinics from 2006 to 2011 for PCOS assessment according to Rotterdam criteria [20]. All protocol procedures were approved by the local ethics committee of the Medical University of Graz (EC-number18-066 ex 06/07). Information about side effects was either collected with a questionnaire during control visits to check for compliance or by phone call.

### 2.2. SNP Selection

#### Discovery Cohort

**Figure 1:**
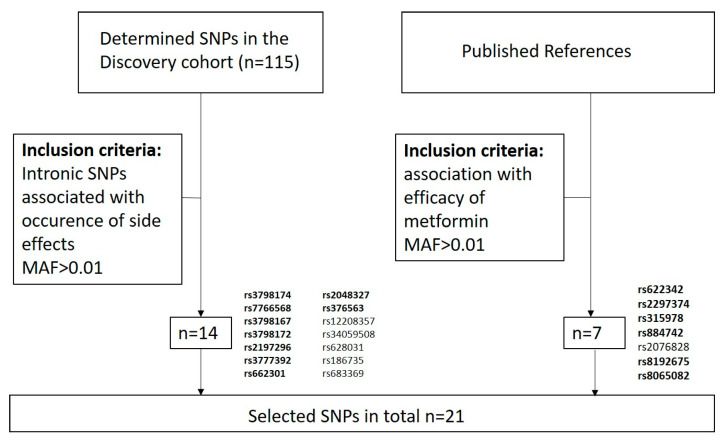
Flow chart of SNP selection. Intronic (non-coding) SNPs are highlighted in bold.

115 intronic SNPs in SLC22A1 (OCT1), SLC22A2 (OCT2) and SLC22A3 (OCT3) with a MAF >0.01 in CEU cohorts were selected for Next Generation Sequencing (targeted SNP sequencing using the Illumina NextSeq) by LGC Genomics GmbH (Berlin, Germany) in 120 participant samples. LD analysis was performed with the LDlink tool of the NIH National Cancer Institute (RRID: SCR_011403, https://analysistools.nci.nih.gov/LDlink/ (accessed on 16 September 2022) [22] and was additionally checked in HaploReg v4.1 (RRID: SCR_006796) [23,24]. The investigated SNPs were not in LD with any coding SNPs. In total, 22 SNPs were chosen for genotyping in the replication cohorts. Inclusion criteria for SNP selection of the discovery cohort was association with occurrence of side effects (*n* = 15). In addition, we selected seven SNPs out of five genes associated with metformin efficacy (*n* = 7) [23,24,25,26,27,28,29] based on previous data and publications. As a further selection criterion, we defined a minor allele frequency (MAF) > 0.1 (Figure 1).

### 2.3. DNA Isolation and SNP Genotyping

Chromosomal DNA was either isolated from EDTA blood or out of serum. Blood samples of the discovery cohort, as well as replication cohort 2, were collected in tubes containing EDTA as anticoagulant. DNA isolation was performed with the NucleoSpin Blood Kit (Macherey-Nagel, Düren, Germany) according to the manufacturer. Isolation of chromosomal DNA from serum samples was performed with the ChargeSwitchTM gDNA Serum Kit (Thermo Fisher Scientific Inc., Bothell, WA, USA) according to the manufacturer for samples of replication cohort 1. DNA quantity and quality was assessed with the QuantiFluor^®^ dsDNA System (Promega GmbH, Walldorf, Austria). Isolated DNA was diluted threefold and 1 µL was used for genotyping with predesigned TaqMan SNP genotyping assays (Thermo Fisher Scientific Inc., Bothell, WA, USA). Endpoint fluorescence was measured with the Fluoroskan Ascent system (Thermo Labsystems, Fischer Scientific GmbH, Wien, Austria). Fluorescence data were analyzed as scatter plots.

### 2.4. Functional Analysis of Intronic SNPs

#### Expression Quantitative Trait Locus Analyses

Examined intronic SNPs were tested for cis-quantitative trait loci (eQTLs) or splice QTLs in any, but particularly in gastrointestinal tissues and the liver, by use of the Genotype-Tissue by the use of the Genotype-Tissue Expression (GTEx RRID: SCR_013042) data release V8 [30,31,32].

*Analysis of chromatin structure changes* was done by analysis with HaploReg v4.1 (RRID: SCR_006796) [33,34]. Changes in transcription factor binding sites and sites of epigenetic modification were determined via HaploReg v4.1.

*Changes in miRNA sequences or their binding sites* were investigated by use of miRdSNP v11.03, Center for Computational Research SUNY at Buffalo [35].

### 2.5. Statistical Analysis

Data are presented as mean ± standard deviation (SD) unless otherwise stated. Nominal variables were analyzed using the χ^2^ and Fisher exact tests. The Shapiro–Wilk test was used to examine for normal distribution. Differences in continuous parameters between genotypes were assessed using analysis of variance (ANOVA) or analysis of covariance, the Mann–Whitney U test, and the Kruskal–Wallis test. Binary logistic regression models were used to determine factors influencing the presence of side effects after metformin intake. A model unadjusted and adjusted to age, concomitant medication, sex and weight according to Dujic et al. was calculated [8]. Due to the low number of homozygous minor allele carriers, they were combined with heterozygous minor allele carriers and compared to homozygous major allele carriers in the analysis performed. A *p*-value < 0.05 was considered significant. A *p*-value > 0.05 and <0.1 was considered as a trend. Statistical analysis was performed using SPSS software version 26 (IBM Corp., New York, NY, USA).

## 3. Results

### 3.1. Discovery Cohort

We included 120 metformin users in our discovery cohort, which consisted of 113 (94.1%) individuals with T2DM, six (5%) women with PCOS and T2DM and one (0.85%) woman with PCOS. Of these, 47 (39%) persons received metformin monotherapy and 73 (61%) metformin in combination with other antihyperglycaemic agents. A total of 17 participants were taking their prescribed dose of 500 mg of metformin, 17 of them were taking 850 mg and 86 persons were using 1000 mg of metformin at the study visit. Due to the lack of information on the side effects of metformin one participant had to be excluded. All investigated parameters did not differ between individuals without metformin side effects and individuals with metformin side effects. Further participant characteristics are given in Table 1.

#### 3.1.1. Gender Specific Side Effects

We investigated the influence on gender-specific side effects in the discovery cohort. Significantly more female study participants reported nausea as a side effect than male participants (*p* = 0.030). All other side effects were reported equally in both genders (Table 2).

#### 3.1.2. SNPs Associated with Metformin Side Effects

Of the 115 genotyped SNPs, 14 showed associations with either all side effects, gastrointestinal side effects or other side effects in a preliminary chi square analysis and were therefore selected for replication (Figure 1). Based on their effect size, we included 13 of these associated SNPs in a binary logistic regression model. Of these, ten SNPs showed associations with side effects in either the unadjusted and/or the adjusted binary logistic regression model. Rs3798167 and rs2197296 were associated with an increased risk for all investigated side effect groups in the unadjusted as well as in the adjusted model. The presence of minor alleles of seven SNPs increased the odds ratio to develop side effects (side effects; four for other side effects) and two SNPs decreased the presence of side effects (one for gastrointestinal and other side effects, respectively, Table 3).

### 3.2. Replication

#### 3.2.1. Reporting on Metformin Side Effects

Replication cohort 1—out of 142 contacted people with T2DM who were treated with metformin in the GIRO study, 30 participants reported side effects from metformin use, while 91 participants reported experiencing no side effects. Twenty-one participants could not be reached by phone.

Replication cohort 2—in the PCOS cohort, 178 women used metformin to improve their symptoms; 126 women experienced no consciously perceived side effects whereas 52 women outlined side effects after metformin intake. A total of 24 women reported diarrhoea; 23 had nausea, no appetite, vomiting or loss of weight. Six reported flatulence, abdominal pain or meteorism. Four women showed symptoms of hypoglycaemia, two had headache or prickling fatigue and one woman reported gain of weight and hair loss. The occurrence of side effects did not significantly differ between individuals with T2DM and women with PCOS (*p* = 0.430). Anthropometric data of both replication cohorts are listed in Table 4.

#### 3.2.2. SNPs Associated with Metformin-Induced Side Effects

Four SNPs from the discovery cohort and one SNP previously associated with metformin efficacy [28] were replicated in either the combined and/or in subgroup analysis.To predict metformin side effects we used linear regression models including the minor alleles of all 22 selected SNPs in one model combined with both replication cohorts and another with each individual. SNPs with significant odds ratios (OR) are shown in Table 5. In the combined model the minor allele of SNPs rs3798167 and rs2197296 significantly increased the OR for the occurrence of metformin side effects, while the minor allele of rs3777392, rs628031 and rs8192675 decreased the OR for metformin side effects (Table 5). In the model with T2DM, the minor allele of rs3798167 increased the OR for the occurrence of side effects and the minor allele of rs3777392 and rs628031 decreased it (Table 5). In the model calculated only with women of the PCOS cohort, the minor allele of rs2197296 increased the risk for the occurrence of side effects and SNPs rs3777392 and rs8192675 decreased it (Table 5).

#### 3.2.3. SNPs Associated with Metformin Side Effects Are Distributed Differently between Individuals with T2DM and Women with PCOS

With the exception of rs2197296, genotype frequencies of metformin side effects associated SNPs differ significantly between T2DM individuals and PCOS women (Table 6).

### 3.3. Polygenetic Risk Score

**Figure 2:**
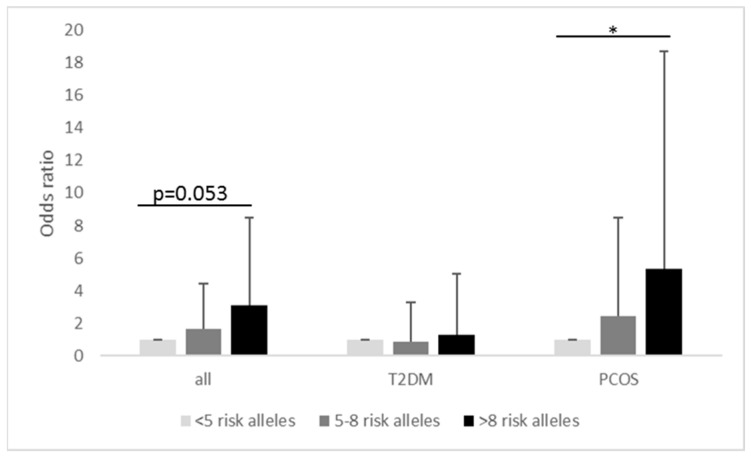
Association of an unweighted polygenetic risk score (PRS) derived from metformin side effects associated with SNPs. Bars indicate 95 percent confidence interval. * *p* < 0.05.

To estimate genetic predisposition for metformin-induced side effects, we evaluated a quantitative unweighted PRS. The PRS was generated by summing the number of risk alleles for each person. The higher the number of risk alleles, the higher the (theoretical) genetic predisposition. Risk alleles comprised the minor alleles of rs3798167 and rs2197296 and the major alleles of rs3777392, rs628031 and rs8192675. Neither in individuals with T2DM nor in women with PCOS were persons with 0, 1 or 2 risk alleles identified. PRS groups were therefore designed as follows: >5 risk alleles (*n* = 25), 5–8 risk alleles (*n* = 177) and >8 risk alleles (*n* = 97). As shown in Figure 2, the OR increases with the number of risk alleles. In all participants the presence of 5–8 risk alleles was not associated with a numerically increased risk of side effects (OR: 1.63 (95% CI: 0.53–5.03), *p* = 0.392), whereas the presence of more than 8 risk alleles by trend led to a more than threefold increased risk to develop side effects OR: 3.09 (95% CI: 0.99–9.75), albeit not reaching statistical significance (*p* = 0.053) (Figure 2).

Subgroup analysis showed no associations of the PRS with side effect development in T2DM (<5 risk alleles *n* = 8; 5–8 risk alleles (*n* = 76) OR. 0.864; 95% CI: 0.160–4.679; *p* = 0.866; >8 risk alleles (*n* = 37) OR: 1.269; 95%CI: 0.221–7.294; *p* = 0.789. In women with PCOS, the presence of >8 risk alleles was associated with a more than five-fold increased risk of developing side effects: (<5 risk alleles *n* = 17; 5–8 risk alleles (*n* = 101) OR: 2.47 (95% CI: 0.53–11.54); *p* = 0.251; >8 risk alleles (*n* = 60); OR: 5.36. (95%CI: 1.12–25.55); *p* = 0.035.

### 3.4. Functional Annotation

Expression Quantitative Trait Locus Analyses and analysis of chromatin structure changes. Annotated SNPs act as eQTLs or sQTLs in different tissues such as whole blood, skin, liver, colon and intestine via different mechanisms. SNPs rs3777392, rs628031 and rs8192675 were replicated at least in one of our cohorts (Table 5). They act as genetic enhancers by altering chromatin structure in the liver and or the mucosa of the duodenum (Table 7). Rs3798167 and rs2197296 replicated SNPs of SLC22A1 and A2 act as eQTLs and modify expressions of OCT3, OCT1 and GLUT2. Rs628031, rs3798167 and rs8192675 act as eQTLs that alter regulatory regions of genes and as sQTLs that affect mRNA splicing and structure.

## 4. Discussion

Based on data from the present discovery cohort of 120 metformin users and the previously described association with metformin efficacy [23,24,25,26,27,28], we investigated 22 mostly intronic polymorphisms in two independent cohorts, one in participants with T2DM and another in women with PCOS (Figure 1). We replicated six intronic SNPs associated with side effects in the combined replication cohort of participants with T2DM and PCOS in a linear regression model (Table 5). In the subgroup analyses of each replication cohort, the association did not appear to be consistent. Heterogeneity of the subgroups in terms of sex, genetic or environmental factors could modulate the effect of the SNPs on metformin induced side effects. It is known that female patients have a 1.5 to 1.7 higher risk of developing adverse side effects compared to male patients [36]. Gender-related differences included perceptual, pharmacokinetic, immunological and hormonal factors, as well as differences in the use of medications by women compared with men, and the reporting on side effects itself. How these differences result in an increased risk of side or adverse effects are not entirely clear [36]. In our discovery cohort, significantly more female study participants reported nausea compared to males. All other side effects were reported equally in both sexes (Table 2). Gender differences in taking metformin or reporting of the side effects could not be excluded. Metformin might exert part of its insulin sensitizing effects through gut microbiota, although currently available data are not consistent [37]. Numerous studies have confirmed that the composition of the gut microbiota in patients with T2DM [38], as well as in women with PCOS [39], is altered. We cannot exclude that disease-related microbiota dysbiosis may contribute to the observed differences in side effects between our two replication cohorts. Rs8192675, a SNP in the SLC2A2 gene, was selected for replication because of its known influence on metformin efficacy. Rathmann et al. published that the C-allele is associated with an improved glucose response to metformin monotherapy during the first year after diagnosis in T2DM [28] and shows in our data as reduced OR for occurrence of metformin side effects (Table 5). It is unclear whether the improved response of hemoglobin A1C (HbA1c) to metformin in the presence of the C variant can be explained by a lower hepatic glucose production or by improved glucose disposal to the liver or peripheral tissues [40].

To generate hypotheses about the mechanistic effects of the replicated noncoding variants on metformin side effects, we performed a functional annotation. Modes of action of intronic SNPs are very diverse. Introns enhance transcript levels by affecting the rate of transcription, nuclear export and transcript stability. Moreover, effects on the efficiency of mRNA translation [41] and on gene expression at the post-transcriptional level are known [42]. SNPs located at introns potentially influence gene expression at all levels mentioned above, as well as via epigenetic changes. We determined possible functional consequences of the selected SNPs by the use of various databases in three main areas: SNPs were tested (1) translationally for eQTLs and sQTLS, (2) epigenetically for changes in chromatin structure and (3) post-translationally for modifications such as changes in binding sites for miRNAs (Table 7), mainly in gastrointestinal tissue and in the liver.

Six SNPs identified in the discovery study (Table 3) were shown to act either as eQTL, sQTL or change chromatin states in gastrointestinal tissue, which might explain their association with gastrointestinal side effects (Figure 2). The investigated SNPs in genes for OCT 1 and 2 were e/sQTLs for genes in the OCT cluster but were predominantly active in the liver, potentially fitting with the lack of associations with gastrointestinal side effects. Four SNPs, e.g., rs3798167, showed, according to our functional annotation, changes in the binding site of transcriptional factors that potentially modulate various genes important for the pharmacokinetics of metformin. Although we cannot derive a definite hypothesis from our functional annotation, it shows that these replicated SNPs are likely to be functionally involved in the development of metformin side effects.

To estimate the genetic risk for developing metformin side effects, we performed a simple unweighted PRS which assigns each genotype the same influence on the total risk score (“allele count model”) [43]. In our cohort, no individual had less than three risk alleles which correspond to the selection criterion of a MAF > 0.01. As expected, the OR increases with the number of risk alleles. All participants with <5 risk alleles did not show a higher risk for the occurrence of side effects (OR: 1.63; 95% CI: 0.53–5.03; *p* = 0.392), whereas participants with >8 risk alleles showed a threefold increased risk of developing side effects, just missing statistical significance (OR: 3.09; 95% CI: 0.99–9.75; *p* = 0.053). It should be mentioned that this score has less predictive power, because (1) it is based on genetic data and does not take gene environment interaction or comorbidities into account and (2) the proportion of genetic variability captured by the set of SNPs is relatively small. This PRS is therefore not suitable as a sole indicator of occurrence of metformin side effects, but is suitable as an additional tool to more accurate risk assessment and to promote future developments to increase the adherence of patients to their medication.

A limitation of this study is the small number of participants in the discovery study, which can lead to false positive results, and the use of two replication cohorts originally not designed to investigate metformin side effects. This does not allow us to specifically focus on gastrointestinal side effects in the analysis of the replication cohorts, although gastrointestinal intolerance represents the most common adverse effect of metformin treatment. Due to the low numbers of metformin intolerant persons in the replication cohort and the high number of SNPs associated with side effects, no analysis of haplo- or diplotypes was performed. We have to mention that in cases of concomitant medications the possibility of a combined effect of these along with metformin cannot be ruled out. Another limitation is that the study participants were taking a relatively low dose of metformin. Further, the investigated SNPs may not be the causal variants but might be in linkage disequilibrium with potential important loci. The strengths of our investigation lie in the in-depth clinical and biochemical characterisation of all participants as well as in the application of a state-of-the-art genotyping method that provides very accurate, reproducible and reliable results.

We conclude that the occurrence of metformin side effects is influenced by polygenic factors including the novel identified intronic SNPs from the OCT cluster. To our knowledge, this work is the first study to specifically address the identification and impact of intronic SNPs on the side effects of metformin. In addition, we are the first to link rs8192675, already known to influence metformin efficacy, with a reduced risk to develop metformin side effects in individuals with disturbances of insulin sensitivity. These new insights are of interest for personalized therapy options.

## Figures and Tables

**Table 1:** Description of study participants in the discovery cohort.

	All	No Metformin Side Effects	Metformin Side Effects
N (%)	119 (100)	71 (59)	48 (41)
age [yr]	59 ± 10	60 ± 10	57 ± 11
BMI [kg/m^2^]	31 ± 5	31 ± 6	31 ± 5
hip circumference [cm]	111 ± 12	110 ± 13	111 ± 11
waist circumference [cm]	105 ± 14	105 ± 14	104 ± 13

Frequency data are presented as number (percentage), continuous data as mean ± standard deviation. N, number; SD, standard deviation; kg, kilogram; m, metres; cm, centimetres.

**Table 2:** Presence of metformin induced side effects in all, male and female study participants.

Symptoms	All (*n* = 119)	Male (*n* = 71)	Female (*n* = 48)	*p*-Value
side effects total	54 (45.8)	30 (42.9)	24 (50.0)	0.459
GI side effects	46 (39.0)	24 (34.3)	22 (45.8)	0.206
diarrhoea	24 (20.3)	12 (17.1)	12 (25.0)	0.354
nausea	9 (7.6)	2 (2.9)	7 (14.6)	**0.030**
flatulence/bloating	33 (28.0)	17 (24.3)	16 (33.3)	0.282
constipation	2 (1.7)	2 (2.9)	0 (0.0)	n.a.
heart burn	1 (0.8)	0 (0.0)	1 (2.1)	n.a.
other side effects	10 (8.5)	6 (8.6)	4 (8.3)	0.964
dizziness	7 (5.9)	4 (5.7)	3 (6.3)	0.904
headache	7 (5.9)	4 (5.7)	3 (6.3)	0.904
dry mouth	1 (0.8)	0 (0.0)	1 (2.1)	n.a.

Frequency data are presented as number (percentage). Significant *p*-values are highlighted in bold. N, numbers; %, percent; GI, gastrointestinal; n.a., not applicable.

**Table 3:** Odds ratios of investigated SNPs in an unadjusted and adjusted binary logistic regression model.

	Unadjusted Binary Logistic Regression Model						Adjusted Binary Logistic Regression Model			
	Side Effects Total OR (95% CI)	*p*	GI Side Effects OR (95% CI)	*p*	Other Side Effects OR (95% CI)	*p*	Side Effects Total OR (95% CI)	*p*	GI Side Effects OR (95% CI)	*p*
**rs3798174**	2.74 (1.05–7.11)	**0.039**	2.52 (0.99–6.39)	0.052	1.8 (0.43–7.58)	0.423	2.91 (1.09–7.76)	**0.033**	2.67 (1.02–6.98)	**0.045**
**rs3798172**	4.05 (1.03–15.84)	**0.045**	2.4222 (0.72–8.18)	0.154	2.325 (0.43–12.49)	0.325	4.54 (1.08–19.06)	**0.039**	2.66 (0.73–9.67)	0.137
**rs3777392**	0.41 (0.17–1.00)	**0.049**	0.49 (0.19–1.21)	0.122	0 (0−n.a.)	0.998	0.51 (0.14–0.90)	**0.029**	0.43 (0.17–1.12)	0.085
**rs35167514**	1.29 (0.86–1.94)	0.224	1.02 (0.68–1.55)	0.911	2.65 (1.30–5.39)	**0.007**	1.79 (0.77–4.17)	0.179	1.07 (0.45–2.55)	0.872
**rs7766568**	2.94 (1.54–21.88)	**0.009**	2.70 (0.89–8.21)	0.080	3.25 (0.74–14.28)	0.119	6.45 (1.63–25.53)	**0.008**	2.85 (0.89–9.07)	0.077
rs3798167 **	2.94 (1.31–6.59)	**0.009**	2.85 (1.27–6.37)	**0.011**	3.38 (0.89–12.79)	0.073	2.76 (1.21–6.27)	**0.016**	2.69 (1.18–6.13)	**0.018**
rs2197296 **	2.25 (1.06–4.79)	**0.035**	2.43 (1.11–5.32)	**0.026**	2.25 (0.55–9.16)	0.260	2.27 (1.04–4.95)	**0.039**	2.50 (1.11–5.60)	**0.026**
**rs9347386**	1.28 (0.59–2.74)	0.531	1.10 (0.50–2.40)	0.813	2.60 (0.53–12.88)	0.241	1.32 (0.59–2.93)	0.499	1.16 (0.51–2.63)	0.719
**rs9347388**	1.40 (0.65–3.01)	0.385	1.20 (0.55–2.61)	0.654	2.75 (0.56–13.62)	0.214	1.43 (0.64–3.16)	0.382	1.24 (0.55–2.80)	0.601
**rs1382785**	1.12 (0.50–2.53)	0.786	1.00 (0.43–2.28)	0.990	2.33 (0.46–11.87)	0.307	1.20 (0.51–2.79)	0.674	1.08 (0.46–2.55)	0.857
**rs662301**	1.23 (0.45–3.38)	0.684	0.76 (0.26–2.21)	0.618	7.00 (1.78–27.52)	**0.005**	1.37 (0.48–3.90)	0.552	0.85 (0.29–2.54)	0.775
**rs376563**	0.38 (0.16–0.92)	**0.033**	0.36 (0.14–0.93)	**0.036**	0.64 (0.13–3.23)7	0.595	0.40 (0.16–0.99)	**0.047**	0.36 (0.14–0.96)	**0.042**
rs2292334 *	0.43 (0.14–1.33)	0.144	0.29 (0.08–1.07)	0.062	1.47 (0.28–7.59)	0.648	0.47 (0.14–1.37)	0.154	0.28 (0.07–1.07)	0.062

* In linkage disequilibrium with rs2048327. ** Associated with an increased risk for all investigated side effect groups in the unadjusted as well as in the adjusted model. OR: odds ratio; *p*: *p*-value; CI: confidence interval; GI: gastrointestinal; n.a.: not available data. Adjustment included age, presence of OCT blocking medications, sex and weight. Significant *p*-values are highlighted in bold. Due to the low number of other side effects and thus decreased power. The adjusted model was not applied to the group of other side effects.

**Table 4:** Characterization of individuals in both replication cohorts.

Replication Cohort 1-GIRO	All Giro Patients (*n* = 121)	No Metformin Side Effects (*n* = 91)	Metformin Side Effects (*n* = 30)
age [years]	62 ± 12	62 ± 12	59 ± 12
height [cm]	172 ± 9	172 ± 9	170 ± 9
weight [kg]	93 ± 20	93 ± 21	90 ± 19
waist circumference [cm]	110 ± 16	112 ± 16	107 ± 16
hip circumference [cm]	110 ± 12	110 ± 11	111 ± 14
Replication Cohort 2-PCOS	All PCOS Patients (*n* = 178)	No Metformin Side Effects (*n* = 126)	Metformin Side Effects (*n* = 52)
age [years]	28 ± 8	29 ± 8	27 ± 7
height [cm]	166 ± 6	166 ± 6	167 ± 6
weight [kg]	84 ± 23	86 ± 23	81 ± 23
waist circumference [cm]	96 ± 20	98 ± 21	91 ± 19
hip circumference [cm]	113 ± 15	115 ± 15	109 ± 14

Continuous data are presented as mean ± standard deviation. cm: centimetres; kg: kilogram.

**Table 5:** Odds ratios of metformin associated SNPs.

SNP	Gene	Combined Metformin Side Effects OR (95% CI); *p*	T2DM Metformin Side Effects OR (95% CI); *p*	PCOS Metformin Side Effects OR (95% CI); *p*
rs3798167	SLC22A1 (OCT1)	**1.68 (1.14–2.49); 0.009**	**2.45 (1.35–4.44); 0.003**	1.35 (0.78–2.34); 0.286
rs2197296	SLC22A1 (OCT1)	**1.93 (1.15–3.23); 0.012**	1.31 (0.57–3.02); 0.521	**2.41 (1.23–4.75); 0.010**
rs3777392	SLC22A1 (OCT1)	**0.34 (0.19–0.61); <0.001**	**0.20 (0.08–0.51); <0.001**	**0.42 (0.17–1-01); 0.048**
rs628031	SLC22A1 (OCT1)	**0.47 (0.25–0.88); 0.016**	**0.34 (0.14–0.85); 0.017**	0.66 (0.27–1.65); 0.372
rs8192675 *	SLC2A2 (GLUT2)	**0.60 (0.37–0.95); 0.029**	1.30 (0.47–3.56); 0.614	**0.56 (0.32–0.97); 0.039**

A linear regression model including the minor alleles of each polymorphism to predict metformin side effects. Significant ORs are highlighted in bold. * Previously associated with metformin efficiency. SNP: single nucleotide polymorphism; OR: odds ratio; 95% CI: 95 percent confidence interval; PCOS: polycystic ovary syndrome; SLC: solute carrier transporter; OCT: organic cation transporter; GLUT2: Glucose transporter 2.

**Table 6:** Genotype distribution of metformin-associated SNPs in participants with T2DM and in women with PCOS.

SNP	Genotype	T2DM *n* (%)	PCOS *n* (%)	*p*-Value
rs3798167	GG	45 (37)	102 (57)	0.007
	GT	55 (45)	59 (33)	
	TT	21 (17)	15 (8)	
rs2197296	AA	0 (0)	0 (0)	0.141
	AG	50 (41)	93 (52)	
	GG	71 (59)	85 (48)	
rs3777392	CC	36 (30)	136 (76)	<0.001
	CT	56 (46)	35 (20)	
	TT	22 (18)	6 (3)	
rs628031	AA	18 (15)	62 (35)	<0.001
	AG	46 (38)	85 (48)	
	GG	54 (45)	31 (17)	
rs8192675	CC	10 (8)	22 (12)	0.003
	CT	34 (28)	65 (37)	
	TT	66 (55)	90 (51)	

Data were compared by χ^2^ test. SNP: single nucleotide polymorphism; PCOS: polycystic ovary syndrome.

**Table 7:** Functional annotation of the replicated SNPs.

SNP	Gene (Protein)	eQTL		sQTL		Chromatin States *		Histone Modifications				Changes in Transcription Factor Binding Sites
		tissue	gen	tissue	gen	type of change	tissue	H3K4me1	H3K4me3	H3K27ac	H3K9ac	
rs3798167	SLC22A1 (OCT1)	various	RP11-288H12.3. OCT3. OCT1	whole blood	OCT1							MIF-1. Mxi1_disc1. Soc: 10. 16. 17. 4
rs2197296	SLC22A1 (OCT1)	various	OCT3. IGF2R. OCT1									MAZ. PRDM1-Known1. Rad21_disc10. Rhox11. STAT_disc7
rs3777392	SLC22A2 (OCT2)			various	OCT1. IGF2R	genic enhancer	liver	small int. duod mucosa liver	liver	liver	liver. duod mucosa	CAC-binding-protein. PU.1_disc3. TATA_disc7
rs8192675	SLC2A2 (GLUT2)	various	EIF5A2. GLUT2. RPL22L1	liver	GLUT2	genic enhancer	liver	duod mucosa. liver		liver	liver	

TSS: transcription start site; duod: duodenum; int: intestine; sigm: sigmoid; skel: skeletal; * core 15 model.

## Data Availability

Restrictions apply to the availability of these data. Data of the PCOS and of the GIRO cohort are available upon request. To get access. a proposal must be submitted to Barbara Obermayer-Pietsch (barbara.obermayer@medunigraz.at) for the PCOS cohort and to Harald Sourij (ha.sourij@medunigraz.at) for the GIRO cohort.
